# Developing and Validating a Primary Care EMR-based Frailty Definition using Machine Learning

**DOI:** 10.23889/ijpds.v5i1.1344

**Published:** 2020-09-01

**Authors:** PhD Tyler Williamson, Sylvia Aponte-Hao, Bria Mele, Brendan Cord Lethebe, Charles Leduc, Manpreet Thandi, Alan Katz, Sabrina T Wong

**Affiliations:** 1 Department of Community Health Sciences, Cumming School of Medicine, University of Calgary; 2 O’Brien Institute for Public Health and Alberta Children’s Hospital Research Institute, Cumming School of Medicine, University of Calgary; 3 Centre for Health Informatics, Cumming School of Medicine, University of Calgary; 4 Clinical Research Unit, Cumming School of Medicine, University of Calgary; 5 Department of Family Medicine, Cumming School of Medicine, University of Calgary; 6 Departments of Family Medicine and Community Health Sciences, Manitoba Centre for Health Policy, University of Manitoba; 7 School of Nursing, University of British Columba; 8 Centre for Health Services and Policy Research, University of British Columbia

## Abstract

**Introduction:**

Individuals who have been identified as frail have an increased state of vulnerability, often leading to adverse health events, increased health spending, and potentially detrimental outcomes.

**Objective:**

The objective of this work is to develop and validate a case definition for frailty that can be used in a primary care electronic medical record database.

**Methods:**

This is a cross-sectional validation study using data from the Canadian Primary Care Sentinel Surveillance Network (CPCSSN) in Southern Alberta. 52 CPCSSN sentinels assessed a random sample of their own patients using the Rockwood Clinical Frailty scale, resulting in a total of 875 patients to be used as reference standard. Patients must be over the age of 65 and have had a clinic visit within the last 24 months. The case definition for frailty was developed using machine learning methods using CPCSSN records for the 875 patients.

**Results:**

Of the 875 patients, 155 (17.7%) were frail and 720 (84.2%) were not frail. Validation metrics of the case definition were: sensitivity and specificity of 0.28, 95% CI (0.21 to 0.36) and 0.94, 95% CI (0.93 to 0.96), respectively; PPV and NPV of 0.53, 95% CI (0.42 to 0.64) and 0.86, 95% CI (0.83 to 0.88), respectively.

**Conclusions:**

The low sensitivity and specificity results could be because frailty as a construct remains under-developed and relatively poorly understood due to its complex nature. These results contribute to the literature by demonstrating that case definitions for frailty require expert consensus and potentially more sophisticated algorithms to be successful.

## Introduction

A frail older person has been described as a slow, weak, and thin individual who appears older than his/her chronological age [1]. Frail individuals have an increased state of vulnerability, and reduced ability to recover following a stressful event which can then lead to disability and mortality [1]. Annually an estimated 250,000 Canadians who die are considered frail [2]. Yet, there is no single generally accepted clinical definition of frailty [3], making screening, identification and potential intervention difficult. The ability for clinicians and decision-makers to identify frailty is important as it is predictive of adverse health events and increased health spending [4-8]. Approximately 5% of the population, many of whom are frail, are considered high users of the healthcare system and account for the majority (60%) of healthcare utilization and costs [9-11]. In part, these hospital costs are due to those who are severely frail (completely dependent for personal care) to terminally ill [12]. As an example, the last years of life for those living in Ontario cost $4.7B annually, comprising 10% of all government-funded health care [13].

The main goals of caring for those who are frail in primary care are to delay increasing severity of frailty, and to improve function and quality of life while avoiding unnecessary admission to hospital or long-term care. Primary care clinicians are well positioned to identify and work with frail individuals. The longitudinal sustained relationships that primary care providers have with many of their patients allow them to recognize the decline of those who are not managing well. However, more work is needed in consistent and accurate detection and reporting of frailty both in clinical practice and at a population level. Identifying and reporting on those who are frail in primary care could enable targeted communications with patients and families and community-based resources in order to improve patient care, patients’ and caregivers’ quality of life and better use of the healthcare system.

Currently only one validated EMR-specific case definition for frailty exists - the electronic Frailty Index [14] which is based on the Frailty Index [15]. The original Frailty Index and the shorter version implemented as the electronic Frailty Index identify “frailty correlates” which map to a set of Clinical Terms Version 3 Read codes. Clinical Terms Version 3 Read codes are clinical terminology codes commonly used in the UK, and contain information on diagnostics, process of care, and medications. The Clinical Terms Version 3 Read codes are only used in the United Kingdom, and do not easily map to other medical ontologies, such as the Systematized Nomenclature of Medicine (SNOMED) [16] or International Classification of Diseases (ICD) [17] codes, which is used in Canadian primary care. Therefore, there is no EMR-specific case definition for frailty that can be used in Canada.

The objective of this work is to develop and validate a case definition for frailty that can be used in a Canadian primary care electronic medical record (EMR) database – the Canadian Primary Care Sentinel Surveillance Network database. This case definition will enable further screening, surveillance, and actionable analytics at the primary care level leading to improvements in the lives of those living with frailty.

## Methods

The Canadian Primary Care Sentinel Surveillance Network (CPCSSN) is a pan-Canadian network of primary care practice-based research networks that collects de-identified information about patients from primary care electronic medical records (EMR) [19]. This rich resource provides a unique view of primary care in Canada and enables public health surveillance through the secondary data collected by this network.

This study uses CPCSSN data from Southern Alberta, with data ranging from January 1, 2008 to December 31, 2016. All procedures were approved by the Conjoint Health Research Ethics Board at the University of Calgary (REB17-0533_REN1). The research was conducted in two phases: 1) reference set development and 2) training the algorithm to the reference set.

### Reference Set Development.

The first step was the development of an appropriate reference set used to train and assess performance of the frailty case definition. For this, CPCSSN sentinels who are also family physicians from the academic Department of Family Medicine (DFM) at the University of Calgary were recruited. This work is closely aligned with the quality assurance priorities of the DFM.

A random sample of 15 of their own patients age 65 years and older was selected for University of Calgary DFM physicians to review. Physicians were instructed to assess each of these 15 patients using the Rockwood Clinical Frailty Scale (CFS) from memory and, if necessary, by reviewing their electronic notes. The CFS uses clinician judgement to assign individuals a score from 1 (very fit) to 9 (terminally ill) [15]. Rockwood et al. (2005) previously assessed interrater reliability between two CFS ratings (i.e. initial scorings done by physicians and those done later by multidisciplinary teams, which had a intraclass correlation coefficient 0.97, p < 0.001) [7]; the same study also showed that CFS is highly correlated (Pearson’s r = 0.80) with other established frailty index tools and has been found to be a valid and clinically important construct [7,15].

The frailty assessment and patient identifying information was collected by clinic staff, stripped of all direct identifiers and provided to the research team using the patient’s CPCSSN ID to facilitate linkage to the previously extracted CPCSSN data for development of the case definition. A sample of 780 patients was anticipated to provide estimates of the sensitivity with a confidence interval width of no more than 10% assuming a sensitivity estimate of at least 80% and equally high specificity. Patients receiving a score of 5 or more by their CPCSSN sentinel family physician were deemed frail as the rating of 5 on the scale indicates that a patient is ‘mildly frail’ whereas a score of 4 is that a patient is ‘vulnerable’.

All 52 CPCSSN sentinel physicians from the University of Calgary DFM participated. Due to a miscommunication between the DFM and the study team, more than 15 patients were randomly sampled for a few sentinels resulting in a final sample of 875 patients in the reference set, with 155 deemed frail (CFS >= 5), and 720 deemed not frail (CFS <= 4). In our sample, 17.7% were classified as frail. Previously published work suggested a frailty prevalence of 22.7% in a Canadian population above the age of 65 using the Frailty Index [18]. See Table 1 below.

**Table 1: Patient Characteristics:** 

	Frail (n = 155)	Not Frail (n = 720)	Total (n = 875)
Percentage Male - n (%) - missing 17	352 (49.7%)	43 (28.7%)	395 (45.1%)
Age - Median (Q1 - Q3)	80 (72 - 85.5)	71 (67 - 77)	72 (68 - 79)
Number of Encounters in The Last Year - Median (Q1-Q3) - missing 17	32 (8 - 84)	48 (10 - 153)	34 (8 - 96)

Comorbidities – missing 130	Frail (n = 142)	Not Frail (n = 603)	Total (n = 745)

COPD - n (%)	71 (11.8%)	34 (23.9%)	105 (14.1%)
Dementia - n (%)	27 (4.5%)	33 (23.2%)	60 (8.1%)
Depression - n (%)	166 (27.5%)	56 (39.4%)	222 (29.8%)
Diabetes Mellitus - n (%)	84 (59.2%)	318 (52.7%)	402 (54.0%)
Epilepsy - n (%)	6 (1.0%)	3 (2.1%)	9 (1.2%)
Hypertension - n (%)	445 (73.8%)	114 (80.3%)	559 (75.0%)
Osteoarthritis - n (%)	270 (44.8%)	71 (50.0%)	341 (45.8%)
Parkinson's Disease - n (%)	3 (1.9%)	1 (0.0%)	4 (0.5%)

### Feature Selection

Patient information within CPCSSN was extracted and transformed into binary outcomes to be considered as potential features for the machine learning algorithm. The following tables within CPCSSN were used in the feature extraction process: billing; problem list (table of common comorbidities); encounter; encounter diagnoses; exam; labs; medication; patient referrals; known risk factors; patient sex. A detailed breakdown of the types of information captured within these tables and the total number of features extracted from these tables are available in Table 3.

Numerical features such as lab values or biometrics, they were binarized according to standardized categories of “high”, “normal”, and “low” according to existing literature (detailed breakdown of how each lab value and biometric was binarized is available in Table 2). See Table 2 below.

**Table 2: Cut-offs for Lab and Measurement Values:** 

Lab Value	Cut-Offs Used	Cut-Off Label
Serum Creatinine (μmol/L)	< 53	Low
Serum Creatinine (μmol/L)	53 - 106	Normal
Serum Creatinine (μmol/L)	> 106	High
Fasting Glucose (mmol/L)	< 3.9	Low
Fasting Glucose (mmol/L)	3.9 - 6.1	Normal
Fasting Glucose (mmol/L)	> 6.1	High
Hemoglobin (g/L)	135	Low
Hemoglobin (g/L)	135 - 180	Normal
Hemoglobin (g/L)	180	High
TSH (mU/L)	0.5	Low
TSH (mU/L)	0.5 - 5	Normal
TSH (mU/L)	> 5	High
Measurement Value	Cut-Offs Used	Cut-Off Label

BMI	< 22	Low
BMI	22 - 30	Normal
BMI	> 30	High
Systolic Blood Pressure (mmHg)	< 90	Low
Systolic Blood Pressure (mmHg)	90 - 140	Normal
Systolic Blood Pressure (mmHg)	> 140	High

Text data which originated from the free-text fields of encounter, encounter diagnosis, and billing tables, was made into features by creating a term document matrix using unigrams.

Billing codes formatted according to the International Classification of Diseases version 90 (ICD-9) within the encounter, encounter diagnosis, and billing tables, the codes were truncated such that only the first 3 characters of each code was used. The first 3 digits contain information on the category of the disease or procedure, thus information on the etiology, anatomic site, and manifestation would not be included (an example being V22, which would include all codes related to normal pregnancy).

Medications prescribed were classified according to anatomical therapeutic chemical (ATC) classification codes within the medications prescribed table, all codes were reduced to the second level, which indicates the therapeutic subgroup (an example being C03, which is the family of diuretics).

Aggregated features were also created for the billing table, encounter diagnosis table, and medications table. Aggregate features based on number of records per patient were: total number of unique records for each patient in the most recent 2 years, absolute change in number of records from year 2 to year 1; relative change in number of records from year 2 to year 1. Aggregated features that encompassed the entire study duration (the most recent 2 years of data from date of latest encounter) were also created: poly-encounter (total number of unique encounter diagnoses for each patient), polypharmacy (total number of unique medications prescribed for each patient), poly-billing (total number of unique billing codes billed for each patient); these features were categorized using the cut-offs of being less than five, between five and ten, and greater than ten.

A total number of 8,046 binarized features were extracted to be considered, and to prevent sparseness in the feature matrix any feature that had a total of 5 or fewer patients was excluded, which resulted in a total of 3,761 features that was used for the machine learning algorithm. Summary of features are displayed in Table 3. All missing value was recorded as 0 for all binarized features. See Table 3 below.

**Table 3: Description of Features Extracted From CPCSSN Tables:** 

Table (number of patients with data for this table)	Features Extracted	Number of Features	Example
Billing (n = 833)	Truncated ICD-9 codes (3 digits) automatically generated by CPCSSN	468	"005", "805", "V49"
Billing (n = 833)	Unique words that have appeared in "diagnosis text" column	1250	"hypotension", "mention", "and"
Billing (n = 833)	Number of average billing entries per patient per year	3	"less than 5", "between 5 and 10", "greater than 10"
Disease Case Indicator (n = 745)	CPCSSN's automatic algorithm for disease detection for: COPD, dementia, depression, DM, epilepsy, hypertension, osteoarthritis, Parkinson's	8	"Hypertension", "DM", "Epilepsy"
Encounter (n = 858)	Unique words that have appeared in "encounter reason" column	2642	"meds", "two", "symptoms"
Encounter (n = 858)	Number of average encounter entries per patient per year	3	"less than 5", "between 5 and 10", "greater than 10"
Encounter Diagnosis (n = 830)	Truncated ICD-9 codes (3 digits) automatically generated by CPCSSN	465	"005", "117", "V82"
Encounter Diagnosis (n = 830)	Unique words that have appeared in "diagnosis text" column	1294	"actinic", "joint", "not"
Encounter Diagnosis (n = 830)	Number of average encounter diagnosis entries per patient per year	3	"less than 5", "between 5 and 10", "greater than 10"
Exam (n = 849)	Waist measurements (large and small), BMI (normal, high, and low), systolic blood pressure (normal, high, and low)	8	"Small waist", "High BMI", "Normal systolic blood pressure"
Labs (n = 817)	4 lab measurements present in first CPCSSN extraction (creatinine, glucose, Hb, TSH)	12	"low creatinine", "low Hb", "high TSH"
Extra Labs (n = 721)	16 lab measurements present in the additional lab extraction (albumin, ALT, AST, calcium, folate, GGT, MCV, alkaline phosphatase, inorganic phosphorus, potassium, total protein, sodium, free T4, urea, vitamin B12, WBC	47	"low albumin", "normal potassium", "high protein"
Medication (n = 827)	Truncated ATC codes (3 characters) automatically generated by CPCSSN	76	"A01", "B05", V07"
Medication (n = 827)	Unique words that have appeared in the "medication name" column	1698	"500", "release", "erbumine"
Medication (n = 827)	Number of average medication entries per patient per year	3	"less than 5", "between 5 and 10", "greater than 10"
Referral (n = 806)	Referrals recorded for each patient	57	"refer for imaging", "referral to diabetologist", "referral to social worker"
Risk Factor (n = 521)	Statuses of alcohol, exercise, and smoking	7	"alcohol current", "smoking unknown", "smoking never"
Patient (n = 858)	Patient sex	1	"male", "female",
Patient age (n = 875)	Patient age as of 2016	1	70, 65

	Total number of unique features:	8046

### LASSO Logistic Regression

Penalized logistic regression using LASSO (least absolute shrinkage and selection operator) with 10-fold cross validation was initially performed to assess baseline performance, and potentially informative features. Grid-search was used to select the optimum lambda value, which was 0.035.

### Machine Learning

**Figure 1: Visualization of Diagnostic Accuracy Using Various Decision Tree Heights:**
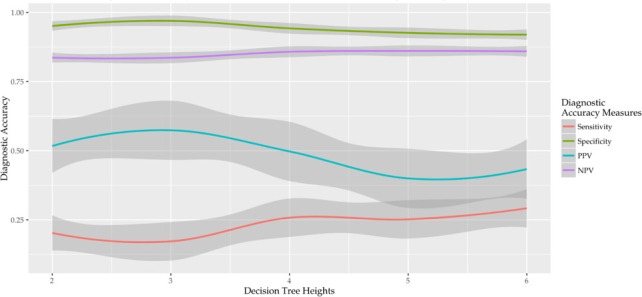


Chi-square Automatic Interaction Detector (CHAID) algorithm, a class of Classification and Regression Tree (CART) supervised machine learning approach was used on the 3,761 features extracted from CPCSSN data for the 875 patients. CART methods were chosen due to their ability to create transparent and interpretable machine learning algorithms best suited for medical diagnostic tools. The hyper-parameters for CHAID was determined by bootstrapping with replacement across combinations from 0.01 to 0.3 by 0.03 for α, maximum decision tree heights from 2 to 6 by 1, and evaluating these hyper-parameters on performances of sensitivity, specificity, PPV and NPV using plots (an example is shown in Figure 1). See Figure 1 below.

The hyper-parameters that resulted in the best overall performance was α = 0.12 and height of 4. 10-fold cross-validation was used to determine the performance metrics. The case definition was considered to have sufficient validity at 70% sensitivity and specificity.

All data analysis was conducted with R Statistical Software version 3.4.2.

## Results

The LASSO logistic regression provided a baseline estimate of how identifiable frailty is using EMR data. The sensitivity was 0.3613, 95% CI (0.2859, 0.4427), specificity was 0.6292, 95% CI (0.5926, 0.6644), PPV was 0.1734 95%CI (0.1346, 0.2201), NPV was 0.8207 95%CI (0.7855, 0.8512).

Key features factored into the CHAID decision tree were: ICD-9 Diagnosis of Dementia (code 290), prescribed medications including: furosemide and vitamins, the key word “obstruction” used within billing notes was also noted. See Table 4 below.

**Table 4: Performance Metrics:** 

Metric	Estimate	95% Confidence Interval
Sensitivity	27.74%	21.01% - 35.60%
Specificity	95.56%	93.71% - 96.89%
PPV	57.33%	45.4% - 68.51%
NPV	86.00%	83.36% - 88.29%
Accuracy	83.54%	80.88% - 85.91%

**Figure 2: Final Decision Tree:**
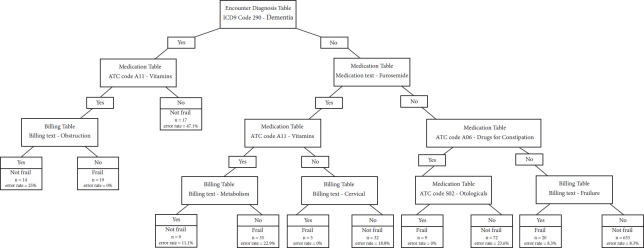


Sensitivity and specificity values were 0.28, 95% CI (0.21 to 0.36) and 0.96, 95% CI (0.94 to 0.97), respectively. PPV and NPV values were 0.57, 95% CI (0.45 to 0.69) and 0.86, 95% CI (0.83 to 0.88), respectively. The performance metrics are detailed in Table 4, and the resulting classification tree is detailed in Figure 2. See Figure 2 below.

## Discussion

### Summary

Currently this is the first to attempt to develop a frailty definition using EMR-derived primary care data in Canada, and the first to attempt to do so using machine learning. The lack of sensitivity, specificity and positive predictive value of the machine learning suggests that identifying frailty using artificial intelligence methods remains complex. Frailty is somewhat elusive and requires additional research to understand the confluence of factors that would identify someone as frail. While clinicians may have a better “gestalt” of whether their patient is frail, a recent systematic review of screening tools for frailty concluded that future research should be targeted at developing a better understanding of what domains are key to defining frailty, with the aim of improving psychometric properties of future screening tools for frailty [19]. That is, instruments measuring frailty may have under-developed construct validity. There is a need to examine frail individuals’ place on the continuum of frailty, of health or death, and where primary care could intervene. This research is a first step towards better understanding of using machine learning to identify the defining features of frailty within the primary care population.

### Explanation

The low sensitivity and specificity of frailty could be due to the fact that frailty as a construct remains under-developed and relatively poorly understood due to its complex nature. There are differing theories about the frailty construct [3, 20-26] and the lack of a clear conceptual definition of frailty is cited as a key reason for instruments’ low diagnostic accuracy properties. Indeed, there are a multiplicity of frailty assessment tools; ten frailty screening tools have been identified for use within primary care settings specifically, and seven screening tools have been applied within geriatric cancer populations [19,27]. Both systematic reviews suggest that none of the available screening tools for frailty have sufficient sensitivity, specificity, negative or positive predictive values. Conversely, unpublished work from Lethebe and Williamson on using machine learning methods for developing CPCSSN case definitions for clearly defined conditions such as diabetes, dementia, epilepsy, and Parkinsonism (conditions with accepted definitions within the literature) have resulted in case definitions with sensitivity values ranging between 95.6% and 98.8% [27].

Some of the identified informative features are consistent with previous literature in its relationship to frailty. Dementia, the first split in the decision tree, has a one-to-one correspondence with frailty in the CFS [28-29], and was shown to have a positive relationship with the Frailty Index (14). The appearance of “vitamins” being prescribed in the medications table may be related to age or nutrition as malnutrition is associated with increased frailty [30], and that nutritional supplementation (including the prescribing of vitamins) has been shown to be a potential therapy for frailty [31-34]. The prescription of furosemide, a common drug prescribed for heart failure was also recorded, and there is evidence to suggest that concurrent heart failure and frailty has been observed in patients and that these patients require targeted interventions [35-37]. Constipation is a prevalent disorder affecting 15% of seniors [38], a risk factor for frailty [39], and is listed as a deficit in the Frailty Index [14]. The identified term “otologicals” relates to hearing loss, as hearing impairment has been reported to associate with frailty [40] and is commonly listed on frailty screening tools for primary care [19].

As the text was processed by single words, it’s difficult to say what single terms such as “obstruction”, “cervical”, and “failure” could mean without the surrounding texts for context. This is a limitation of the methods and could be improved in future analysis by using more advanced language processing methods.

Frailty as a descriptive syndrome must be further defined and understood to aid in detection. This is a fundamental issue that needs to be carefully considered before the utility of a frailty case definition can be truly recognized. This is further complicated by the considerable variability in the manifestation of frailty, partly due to the host of underlying causes. The variability and known identifiers of frailty (e.g. grip strength) may not be captured within the context of EMRs within primary care, as there are no specific medications or billing code associated with frailty in Alberta, unlike more well-defined diseases.

### Limitations

While EMRs are often excellent sources of data on patients in family medicine clinics, there are some limitations to their use in creating an automated case definition. The data available in CPCSSN consist only of a subset of what the physician has recorded. Aspects of a patient’s history may be sparsely recorded, incomplete, and/or difficult to interpret. Additionally, while patient referrals are available to CPCSSN, correspondence between the physician and place of referral are not extracted. Therefore, the outcome of the referral appointment is not available in CPCSSN unless the physician entered in such information in the EMR itself.

Frailty status is also best characterized on a continuum, but this study defined patients as either frail or not frail by selecting a cut-off of 5 using the CFS score, which may have contributed to the difficulties of the case definition in separating frail patients from non-frail patients when the differences between patients with a CFS score of 4 and 5 is very different from those patients whose scores are 1 and 9. Future analysis should consider using the ordinal nature of the CFS scale to best describe the progressive nature of frailty.

No test of interrater reliability was conducted between the different physicians in the reference set development stage. Currently, an unknown proportion of the variability between the frail patients in the study sample can be attributed to different physicians, as the number of patients per physician is too small to determine this. Future studies should consider adding a test of interrater reliability for frailty across all physicians, if the reference standard used arises from physician diagnoses.

## Conclusion

In conclusion, CPCSSN case definitions may be most beneficial for use in cases where conditions have conceptual clarity and are therefore clearly defined. However, the potential to expand upon methodologies applied within this field to account for the non-binary nature of frailty is now understood and will be applied in future research. Frailty is a complex condition that may present in a variety of ways, this research has highlighted the potential for EMRs to account for these features. This will be important in a health care system with a growing aging population. These results contribute to the literature by demonstrating that more heterogeneous diseases may be challenging for machine learning methods as the cases do not necessarily have the same presentation. Some characteristics of frail patients may not be entirely captured within EMRs, thus precise and consistent definitions are required for reference standard creation, and careful refinement is needed for the creation of features for any machine learning algorithm used.

This study contributes to the current epidemiologic knowledge of frailty as well as provides insight into how such cases are and should be recorded in EMRs.

## Acknowledgments

None to declare.

## Ethics statement

The study was reviewed by the University of Calgary Conjoint Health Research Ethics Board (REB17-0533).

## Abbreviations

CPCSSN	Canadian Primary Care Sentinel Surveillance Network
EMR	Electronic medical record
DFM	Department of Family Medicine
CFS	Clinical Frailty Scale
ICD	International Classification of Diseases
ATC	Anatomical therapeutic chemical
CHAID	Chi-square Automatic Interaction Detector
CART	Classification and Regression Tree
PPV	Positive predictive value
NPV	Negative predictive value
